# Current strategies in the management of dementia with lewy bodies and future directions based on disease pathophysiology

**DOI:** 10.1186/s13024-025-00856-7

**Published:** 2025-07-01

**Authors:** Daniel Erskine, John-Paul Taylor

**Affiliations:** Translational and Clinical Research Institute, Biomedical Research Building, Newcastle University, Campus for Ageing and Vitality, Newcastle upon Tyne, NE4 5PL UK

**Keywords:** Dementia with lewy bodies, Alpha-synuclein, Dementia, Neurodegeneration

## Abstract

Dementia with Lewy bodies (DLB) is thought to be the second most common form of dementia after Alzheimer’s disease, and is characterised by a combination of cognitive, neuropsychiatric and motor symptoms. The present review seeks to discuss current strategies for the management of DLB, and future directions for novel disease-modifying therapies. Current best practice for the clinical management of DLB is based upon therapies that target specific symptom domains due to the lack of disease-modifying therapies. Cholinesterase inhibitors are the frontline treatment for treating cognitive decline in DLB, whereas the treatment of motor symptoms remains challenging due to poor response to dopaminergic therapies and the potential for exacerbation of neuropsychiatric features. There is emerging evidence suggesting a range of further pharmacological and non-pharmacological therapies may be effective in treating specific symptom domains of DLB, but further evidence is warranted to demonstrate their efficacy. A key challenge in the treatment of DLB is incomplete understanding of disease pathophysiology, which has limited attempts to develop disease-modifying therapies. In the present article, we discuss the multi-faceted nature of DLB neuropathology, from Lewy body pathology to mitochondrial dysfunction, and discuss therapies in development that target particular aspects of DLB neuropathology. In particular, we highlight antibody-based therapies to attenuate protein aggregation, compounds that enhance the generation of cellular energy and autophagy-enhancing agents as particular areas of promise. Furthermore, we discuss how optimal strategies for disease modification will be centred on agents that treat DLB neuropathology more holistically, and will be underpinned by a more complete understanding of the pathogenic events that underlie the full spectrum of pathological changes observed in the DLB brain.

## Background

Dementia with Lewy bodies (DLB) is a significant and important cause of late life neurodegenerative dementia. It is neuropathologically characterised by the accumulation of the protein α-synuclein into spherical intraneuronal deposits termed Lewy bodies [1] and sits under the umbrella term of Lewy body disease which also includes individuals with Parkinson’s disease (PD) and Parkinson’s disease dementia (PDD). DLB and PDD overlap patho-aetiologically and from a treatment perspective; as clinical syndromes they are currently differentiated arbitrarily on the basis of the temporal onset of cognitive symptoms: PDD is a neurocognitive disorder that occurs in the context of established PD whereas DLB presents with cognitive impairment and the parkinsonism occurs contemporaneously or subsequent to cognitive impairment [2]. In the present review we focus primarily on DLB but take an agnostic perspective on either lumping or splitting these clinical syndromes instead including discussion where appropriate on both conditions where there may be a common aetiological or therapeutic perspective. This approach is also salient given recent efforts to accommodate both PD and DLB under a common biological framework which we discuss further below.

Although clinical estimates are thought to under-estimate the prevalence of DLB, neuropathological studies typically estimate the prevalence of DLB to be approximately 15–20% of all dementia cases and recent data using synuclein amplification assays (SAA) have suggested that abnormal α-synuclein is present in up to 23% of memory clinic patients with cognitive impairment [3, 4].

The clinical features of DLB are not the focus of the present review as they have been covered extensively in international consensus guidelines [2]. Briefly, DLB is a heterogeneous disorder associated with cognitive, motor and neuropsychiatric features. The cognitive profile of DLB typically implicates attention, executive function and visuospatial abilities, and episodic variations in attention and awareness over minutes or hours, a phenomenon known as cognitive fluctuations, are a core feature of DLB [2]. Complex visual hallucinations consisting of well-formed figures, such as animals or people, are another core feature of DLB, present in up to 80% of patients and are an especially distinctive feature of DLB relative to other forms of dementia [2, 5]. Motor features reminiscent of PD, including bradykinesia, rigidity and tremor, are also a core feature of DLB though they tend to be more symmetrical than in PD and respond less well to dopaminergic therapies [6, 7]. REM sleep behaviour disorder, the acting out of dreams during REM sleep is a further core feature of DLB and is thought to precede the onset of DLB by several years [8]. Other features associated with DLB include autonomic dysfunction and neuropsychiatric symptoms such as depression, anxiety and apathy.

The present article aims to summarise the current state of the art regarding the diagnosis and clinical management of DLB, in addition to emerging evidence regarding the pathophysiology of DLB and how these may inform the therapeutic management strategies of the future.

## Main body

### Diagnosis of DLB

International consensus diagnostic criteria take a predominantly clinical approach to the diagnosis of DLB and considers four core clinical features which strongly support a diagnosis of DLB along with a wider range of supportive clinical features which increase the likelihood of the diagnosis of DLB, although in themselves are not as specific (Table 1). As diagnostic criteria have iteratively developed, there has been increasing clarity on the inclusion of biomarkers and the most recent version of the criteria include both indicative (strongly favouring a diagnosis of DLB) and supportive (helping support a diagnosis of DLB but have less diagnostic specificity) biomarkers [2].

**Table 1:** Core and supportive clinical features of DLB [2]

Core clinical features	Description
Fluctuating cognition	Variations in attention and awareness
Visual hallucinations	Recurrent and well formed visual hallucinations
REM sleep behaviour disorder	Acting out dreams
Parkinsonism	One or more of the following: bradykinesia, rest tremor or rigidity
*Supportive clinical features*	*Description*
Neuroleptic sensitivity	Severe sensitivity to antipsychotics, particularly neuroleptics
Autonomic dysfunction	Constipation, orthostatic hypotension, urinary incontinence
Additional features	Falls, syncope, hypersomnia, hyposmia, hallucinations in other modalities, delusions, apathy, anxiety, depression

Refinements to the clinical diagnostic criteria have increased sensitivity and specificity and there is also research criteria for prodromal DLB [8]; however, in clinical practice there is considerable under-diagnosis and under-recognition of DLB [9]. Nevertheless, although there is considerable clinical under-recognition of DLB, the implementation of the consensus diagnostic toolkit has been demonstrated to increase diagnostic rates of DLB but there continues to be a significant mismatch between the expected prevalence of DLB and the proportion of dementia cases receiving such a diagnosis [10]. Therefore, there continues to be a pressing unmet need to develop diagnostic biomarkers that enable measurement of the underlying pathology of DLB to improve clinical recognition of DLB.

SAA represent a potential paradigm shift in how we diagnose synucleinopathies, including DLB. SAA can identify α-synuclein pathology using bodily fluids, based on the capacity of disease-associated α-synuclein to induce the misfolding and aggregation of the native protein in significant abundance to be detectable using fluorescent dyes such as thioflavin [11]. The ability to detect disease-associated α-synuclein pathology in bodily fluids using SAA creates the possibility to detect neuropathology *intra vitam*, overcoming the considerable limitation of neuropathological assessment being the only method to definitively confirm the presence of cerebral α-synucleinopathy. Furthermore, SAA provides a more proximal diagnostic biomarker of the key pathology of abnormal synuclein in contrast to DaT scan or MIBG imaging which are indirect biomarkers of the neurodegenerative impact of synuclein disease and which may be moderated by other pathologic processes. It is notable that recent data using SAAs have suggested that abnormal α-synuclein is present in up to 23% of memory clinic patients with cognitive impairment which is aligned with neuropathological estimates of the prevalence of DLB to be approximately 15–20% of all dementia cases [3, 4].

The effectiveness of SAA to detect α-synucleinopathy, coupled with the central role ascribed to α-synuclein pathology in DLB, has led to the recent development of biological frameworks to define Lewy body diseases anchored primarily on the presence of seed-competent α-synuclein on SAA [12, 13]. Aside from the fact that such frameworks typically subsume DLB into a wider identity centred around PD, the ability of SAA to identify distinct disease states is predicated upon seed-competent α-synuclein pathology being relatively specific for primary Lewy body diseases; however, Lewy body pathology is a common feature of other conditions, particularly Alzheimer’s disease, where it occurs in over 50% of cases [14]. Our studies in *post-mortem* tissue of paediatric lysosomal storage disorders cases have identified seed-competent α-synuclein pathology that gives a positive response on SAA, despite these disorders resulting from the accumulation of lipids rather than proteins [15–17]. Furthermore, recent data from a large cohort of clinically diagnosed DLB cases (*n* = 191) found that CSF α-synuclein SAA was not evident in 28.3% of the sample [18]. Further work clearly is needed to clarify the meaning of these findings whether this is due to loss of sensitivity from an analytic perspective, or is something that relates to the intrinsic sensitivity of SAA to detect brain alpha-synucleinopathy when present in lower abundance or localised to particular brain regions [19]. Clinical misdiagnosis in cohorts of DLB is another possibility and it may be that SAA is more sensitive for the detection of particular subgroups of DLB with more evident parkinsonism, REM sleep symptoms and worse hyposmia [18]. Therefore, whilst SAA is likely to be an important tool for the diagnosis of DLB in future, further validation will be needed especially against neuropathologically confirmed cohorts and it seems likely that, practically, its use will be in combination with existing clinical assessment toolkits and supportive biomarkers.

### Management of DLB

The evidence base for the management of DLB has been covered elsewhere [20]. DLB management is primarily symptomatic, in part, to the current lack of disease-modifying therapies. Pharmacological treatment for DLB typically is primarily focused on the management of cognitive and neuropsychiatric features with acetylcholinesterase inhibitors, such as donepezil, rivastigmine and galantamine. This class of drugs are based on ameliorating cholinergic deficits in DLB patients, which are thought to be the result of degeneration of the primary cholinergic nuclei in the brain of DLB patients, such as the nucleus basalis of Meynert and the pedunculopontine nucleus [21–23]. Acetylcholinesterase inhibitors have generally been demonstrated to give significant, albeit modest, benefits in improving cognitive function and reducing hallucination severity in DLB patients; these effects may be sustained and associate with increased survival [24–26].

The NMDA receptor antagonist, memantine, has also been trialled in DLB. The prevailing mechanistic view has been that NMDA antagonism for DLB is predicated on attenuating excitotoxicity resulting from abnormalities in glutamatergic neurotransmission that leads to a state of excitotoxicity. Other secondary mechanisms of action have been proposed including, for example, cholinergic and dopaminergic modulation, as well as synaptic NMDA actions although how these translate into clinically meaningful impacts on symptoms still remains unclear [27]. Memantine has been demonstrated to be well-tolerated but with only a modest impact on cognition and behaviour and evidence for its efficacy is mixed [28–30].

Neuropsychiatric features, including visual hallucinations, apathy, and delusions, are especially pronounced in DLB compared to other neurodegenerative disorders, and have been demonstrated to have a particularly marked effect on patient quality of life and caregiver burden [31]. Management of neuropsychiatric features is typically targeted and weighed against the potential for adverse events; anti-psychotic medications can induce a severe and even fatal reaction in DLB patients and are thus typically only used with caution and as a second line treatment [20]. Quetiapine is often used given it has the least side effects although evidence for its efficacy is lacking [32]. Clozapine has an evidence base in PD psychosis but has not been trialled in DLB and may come with unacceptable side effects in older frail patients. It is important to emphasise that some antipsychotics should generally be avoided in DLB patients, such as risperidone, olanzapine and aripiprazole, due to risk of deterioration, including increased parkinsonism [33]. Aripiprazole may help neuropsychiatric symptoms but evidence is based on case series also carries attendant risks similar to other antipsychotics for DLB [34, 35].

Pimavanserin, a serotonin inverse agonist has been shown to be more effective and better tolerated than quetiapine in the treatment of hallucinations, delusions and paranoia but is not widely available outside the United States [36, 37]. Benzodiazepines are best avoided in the management of DLB due to their exacerbation of functional decline although clonazepam is sometimes used to manage significant REM sleep behaviour symptoms [38]. Melatonin is sometimes recommended first line for REM sleep behaviour symptoms given its better side effect profile compared to clonazepam; however there is no trial evidence to support its efficacy in DLB [39].

Daytime drowsiness is common in DLB. First line, good sleep hygiene is advocated. Psychostimulants like armodafinil have been trialled in small studies and might help but carry potential side effects and more research is needed to recommend them. Other issues, such as sleep apnoea, may respond to continuous positive airways pressure treatment and should be assessed for by specialist sleep services.

Although antidepressants have been used in the clinical management of DLB relatively few studies have investigated their efficacy, though existing evidence from a systematic review indicates the serotonin selective reuptake inhibitor (SSRI), citalopram, may lead to deterioration of neuropsychiatric features whilst an alternative SSRI, paroxetine, has shown some benefits in case studies [40]. However, given that paroxetine has significant anti-cholinergic activity, caution should be exercised in its use in DLB [41].

The management of motor symptoms in DLB can be challenging, primarily due to DLB patients responding less consistently and effectively to dopaminergic therapies than PD cases, combined with the risk of exacerbating neuropsychiatric features, in particular, hallucinations [42, 43]. Therefore, when dopaminergic therapies are used, they should be at the lowest effective dose and combined with careful monitoring of the patients for deterioration of neuropsychiatric symptoms. Levodopa monotherapy is generally preferred over dopamine agonists due to the view that dopamine agonists may exacerbate neuropsychiatric features of DLB [44]. More recently the anti-convulsant drug zonisamide has been recommended as an adjunct treatment in DLB. This drug has complex pharmacodynamics that includes dopamine agonism, has, in double blind RCTs, demonstrated efficacy in improving parkinsonism in DLB without significant worsening of neuropsychiatric features [45, 46].

Dysautonomia, including but not limited to, orthostatic hypotension, urinary urgency, constipation, etc. has significant impacts on the quality of life of people living with DLB [47]. Unfortunately, there is a paucity of research and therapeutic trials in DLB in this key symptom domain and evidence for the management of these symptoms typically draws on work in PD; coverage of this is beyond the scope of the current review although we would draw the reader’s attention to other reviews and guidelines provide advice on how to manage autonomic symptoms in DLB [39].

The past decade has heralded an increase in studies evaluating the efficacy of non-pharmacological interventions in DLB. Although evidence continues to be collected as to the efficacy of such interventions, most trials to date have been performed in individuals with PDD, where interventions such as cognitive rehabilitation and physical therapy have led to improvements in cognitive and motor symptoms, respectively [48]. Studies in DLB, whilst frequently reporting benefits are often small in sample size and low quality which makes it difficult to make specific non-pharmacological recommendations [49].

Electroceuticals have been advocated as another potential therapeutic route [50]. There have been tentative open label findings of improvements with transcranial direct current stimulation (tDCS) in attention and visuoperceptual function and a small case study (*n* = 6) suggesting transcranial magnetic stimulation may ameliorate depression [51]. However, two randomised sham-controlled trials of transcranial direct current stimulation in DLB and PDD patients did not demonstrate any significant improvements in cognition or hallucinations [52, 53]. Electroconvulsive therapy may help with depression and psychosis in DLB although data is limited to case series [54, 55]. Deep brain stimulation of cholinergic nuclei has also been evaluated in DLB with no consistent improvements in cognitive measures although 3/5 patients treated have a reduction in neuropsychiatric symptoms [56]. Further work is needed to evaluate this approach, however.

Overall, the evidence base for symptomatic management in DLB is piecemeal with the strongest evidence for cholinesterase inhibitors and decreasing levels of evidence for other pharmacological and non-pharmacological approaches. This relative lack of evidence should, however, not abrogate a focus on treating symptoms in DLB; holistic care approaches which take a multi-symptom perspective, for example the Diamond-Lewy management toolkit appear to have demonstrable benefits for patients and their care-givers in global outcomes and in reducing care-giver burden and depression [10]. Critically, going forward, however, is the urgent need to develop drugs which modify the underlying disease process and thus the disease course and trajectory. In the next section, we will discuss the pathophysiology of DLB and expand upon how further understanding of disease aetiology may foster a new era of disease-modifying therapies.

### The pathophysiology of DLB: α-synuclein

The underlying cause of DLB is not known; however, the accumulation of the protein α-synuclein is thought to play a central role [1]. α-synuclein has been implicated in Lewy body diseases as duplications/triplications in its gene, *SNCA*, are associated with autosomal dominant PD, variants in *SNCA* increase risk of idiopathic and familial PD, drugs that repress α-synuclein transcription are associated with reduced risk of PD and α-synuclein is a major component of Lewy bodies [57–60]. Lewy bodies, large spherical deposits immunoreactive for α-synuclein, are the characteristic neuropathological feature associated with DLB. Widespread Lewy body pathology is necessary for a neuropathological diagnosis of DLB, which has underpinned the assumption that they play a central role in disease pathogenesis [1, 2].

Despite the focus on Lewy bodies in DLB, even regions with substantial cell loss, such as the substantia nigra, do not evidence associations between the abundance of Lewy bodies and the degree of neuronal loss or phenotypic severity [61, 62]. Furthermore, we have previously reported neuronal loss in regions of the thalamus with no Lewy body pathology, in contrast to preserved neuronal populations in adjacent regions with Lewy bodies, and others have reported neuronal loss and/or molecular alterations in neurons without Lewy bodies, suggesting a poor relationship between regions that manifest Lewy body pathology and those with neuronal loss [63–65]. The original staging scheme for Lewy body pathology, devised by Braak and colleagues, assumed that neocortical Lewy bodies would associate with clinical dementia; however, this work was performed without clinical data and subsequent studies have demonstrated significant numbers of PD cases with neocortical Lewy bodies but no evidence of clinical dementia, and vice versa [66]. Taken together, despite continued focus on Lewy bodies as central drivers of DLB and other Lewy body diseases, there is little neuropathological or molecular evidence to support an association with neuronal loss or phenotypic severity, in Lewy body diseases.

It is important to note that poor associations between Lewy bodies and meaningful clinical or neuropathological variables does not exclude the potential for α-synuclein to be a central driver of DLB. It has been suggested that a poorly-defined group of multimers that may precede the formation of higher-order α-synuclein aggregates, typically termed “oligomers”, could be the primary toxic agent in DLB and related Lewy body diseases [1]. Novel methods, such as α-synuclein proximity ligation assay, have been employed to detect oligomeric α-synuclein, and typically identify pathology not observed with standard α-synuclein immunohistochemistry [67]. There is limited evidence that α-synuclein oligomers may be better correlates of cognitive dysfunction in DLB than Lewy body pathology; however, this is an under-studied area and further studies are required to better characterise and define oligomers, and evaluate their role in DLB [68].

α-synuclein is extensively post-translationally modified via phosphorylation and truncation and, in DLB, there is a particular increase in the abundance of α-synuclein phosphorylated at serine 129 (pS129) [69]. The precise role of pS129 is not clear, with studies ranging from those demonstrating a physiological role in regulating excitation/inhibition in the neuronal pre-synaptic terminal to a potentially protective role by inhibiting aggregation [70, 71]. Although a number of other post-translational modifications beyond pS129 have been described for α-synuclein, some of which appear to regulate α-synuclein aggregation in vitro, the extent to which these occur in individuals with DLB and thus play a role in disease remains unclear [72].

Genome-wide association studies indicate *SNCA* variants are associated with risk of DLB, and broadly suggest overlap with PD (*GBA1* and *TMEM175*) and Alzheimer’s disease risk genes (*APOE* and *BIN1*) [73]. The overlap with Alzheimer’s disease and PD corresponds well with the neuropathology of DLB, where significant levels of concomitant Alzheimer-type pathology are a frequent observation [74, 75]. The levels of concomitant Alzheimer-type pathology vary widely across DLB cases, typically lying at an intermediate stage based on consensus criteria for the neuropathological reporting of Alzheimer’s disease; however, up to 30% of DLB cases have a high level of Alzheimer-type neuropathological change, a level sufficient for a neuropathological diagnosis of Alzheimer’s disease [74, 76, 77]. The presence of Alzheimer-type pathology in DLB associates with a more amnestic cognitive profile, more rapid disease progression and higher mortality, suggesting it is not an epiphenomenon but a key contributor to phenotypic severity [78]. Therefore, although Lewy body pathology defines DLB, Alzheimer-type pathology is a common concomitant feature that is associated with a worse prognosis.

In summary, despite the important role ascribed to Lewy bodies in DLB, there is relatively little evidence that they are associated with the neurodegeneration and impairment that is thought to underlie clinical manifestation and progression, perhaps suggesting oligomers or other α-synuclein conformers could be more centrally involved in driving DLB. However, there is clear evidence that Alzheimer-type pathology is a common feature of DLB, where it is associated with accelerated decline and early mortality.

### The pathophysiology of DLB: beyond proteopathy

Although there is considerable focus on the role of α-synuclein in DLB, a number of other pathological changes also occur in DLB that may contribute to the symptoms experienced by patients. Cells have a number of mechanisms to prevent the accumulation of misfolded proteins such as α-synuclein, including degradation via the ubiquitin-proteasome system or the autophagy-lysosome system [79]. However, when the accumulation of misfolded proteins cannot be controlled via these processes, misfolded proteins are trafficked through the microtubule network to the perinuclear microtubule organising complex (MOC) and formed into an aggresome, a perinuclear structure to facilitate the containment and degradation of misfolded proteins via the chaperones and proteases present within the MOC [80]. A key outstanding question is why these multiple lines of defence against misfolded proteins go awry in Lewy body diseases, and whether this is indicative of more significant and widespread cellular impairment than the misfolding of α-synuclein in isolation.

Mitochondrial dysfunction has long been implicated in Lewy body diseases, primarily based on evidence from the PD literature demonstrating pathogenic variants in mitochondrial genes, particularly those involved in mitophagy, underlie familial forms of PD [81]. There is much less literature reporting mitochondrial dysfunctional in DLB specifically, though transcriptomics studies have indicated mitochondrial dysfunction and oxidative stress in *post-mortem* DLB brain tissue [82]. Furthermore, we and others have reported reduced expression of components of complex I of the mitochondrial respiratory chain in neurons of the nucleus basalis of Meynert and substantia nigra in DLB [22, 83]. A key question is whether mitochondrial dysfunction is a contributor to DLB or the consequence of another insult, such as α-synucleinopathy. We have previously reported Lewy body pathology is common in the brains of older individuals with primary mitochondrial diseases, suggesting that mitochondrial dysfunction and age predispose towards the development of Lewy body pathology [84]. However, our previous neuropathological observations in individuals with primary mitochondrial diseases demonstrates the striking vulnerability of regions that are resilient to neurodegeneration and Lewy body pathology in DLB, such as the cerebellum and primary visual cortex, perhaps arguing against a primary mitochondrial aetiology for DLB [65, 85, 86]. Oligomeric α-synuclein has been reported to impair complex I-dependent respiration and alter mitochondrial membrane permeability in vitro, suggesting α-synuclein pathology directly impairs mitochondrial function [87, 88]. Taken together, mitochondrial dysfunction is associated with DLB though it remains unclear if it is a cause or consequence of the primary disease process.

Inflammation has also been reported to occur in DLB, primarily due to observations in model systems [89]. Studies in patient-derived plasma suggest an increase in inflammatory cytokines in DLB, particularly early-stage cases, though the extent to which peripheral plasma is a proxy of cerebral changes is debatable, and *post-mortem* evaluation of DLB brain tissue does not typically indicate evidence of inflammation [90, 91]. It is tempting for one to speculate that DLB may be marked by an early inflammatory reaction that subsequently subsides over time, perhaps due to an infection precipitating disease onset. Previous studies have identified that α-synuclein has anti-microbial properties and is significantly up-regulated in conditions of cerebral infection, perhaps suggesting a mechanism underlying early inflammation in DLB that subsides over time [92–94]. Therefore, inflammation continues to be a topical area of research and is frequently posited as a therapeutic target for DLB [91].

The autophagy-lysosomal pathway has been implicated in Lewy body diseases more broadly, primarily as a number of genes encoding proteins involved in the autophagy-lysosomal pathway are associated with risk of PD and DLB [95]. DLB cases manifest alterations in autophagy markers in plasma from DLB cases, and the accumulation of autophagosomes and autophagic mitochondria in DLB neurons, consistent with deficient autophagy in DLB [96, 97]. Studies characterising the ultra-structure of Lewy bodies report they contain apparently damaged organelles which, combined with the observation that Lewy bodies are immunoreactive for markers of the regulated encapsulation of misfolded proteins, aggresomes, is consistent with the sequestration of waste into Lewy bodies as a response to deficient autophagic clearance of cargo [98, 99]. Many risk genes for DLB are specifically involved in the catabolism of a class of lipids termed sphingolipids, the best known of which is *GBA1*, which encodes the lysosomal glucocerebrosidase [73, 100]. We have also previously reported α-synuclein with putative pathological properties in the brain tissue of children with Krabbe disease and metachromatic leukodystrophy, sphingolipid storage disorders caused by Lewy body disease risk genes, suggesting a direct link between deficient sphingolipid homeostasis and α-synuclein aggregation [16, 17]. It is not clear how dysfunction of the lysosomal enzymes encoded by these genes could contribute to the disease process of DLB and related Lewy body diseases; however, we have previously hypothesised that this could reflect the combination of deficient lysosomal clearance and the accumulation of lipid substrates that cross-seed α-synuclein aggregation [101]. It is notable that dyshomeostasis of sphingolipids has previously been reported to underlie the diversity of pathological processes implicated in Lewy body disease, such as deficient autophagy, inflammation and mitochondrial dysfunction, and thus altered sphingolipid metabolism could provide a parsimonious explanation for the range of pathological processes reported in the brain in Lewy body disease [102, 103]. However, further functional work is necessary to understand the relative contribution of distinct pathological processes of DLB more broadly, and multi-disciplinary studies evaluating the full spectrum of pathological changes in DLB are likely to be fruitful in developing therapies that treat the disorder, rather than specific facets of it.

### New horizons in the therapeutic management of DLB

The considerable body of work aiming to understand the pathogenesis of DLB, as summarised above, is likely to open up new fronts in the development of disease-modifying therapies for DLB. To some extent, such efforts have already begun with the development of therapies aiming to ameliorate α-synuclein aggregation, albeit in the context of PD, such as prasinezumab; however, this therapy has not shown meaningful benefits in a Phase II clinical trial beyond an exploratory analysis in a post-hoc sub-group analysis [104, 105]. It is striking that relatively few clinical trials are underway targeting α-synuclein given the central role ascribed to it in the pathogenesis of Lewy body diseases, even in the relatively more active therapeutic pipeline of PD [106]. In part, this may be due to the typically intracellular nature of α-synuclein pathology in contrast to the primarily extracellular amyloid-β, where a number of antibodies have been developed for Alzheimer’s disease [107]. Nevertheless, further therapies targeting α-synuclein aggregation seem a likely development in Lewy body disease in the future, and given the prominent role ascribed to this protein in DLB it seems prudent to evaluate their efficacy in DLB in addition to PD.

As noted above, α-synuclein pathology is not the only proteopathic lesion typically identified in DLB brains, and concomitant Alzheimer-type pathology is also a frequent occurrence and appears to contribute to accelerated cognitive decline. The relatively recent development of antibody-based therapies targeting amyloid-β have demonstrated a remarkable capacity to reduce the burden of cerebral amyloid-β pathology [108, 109]. Given the association between concomitant Alzheimer-type pathology and accelerated cognitive decline in DLB, amyloid antibodies would seem to be a logical option for future evaluation regarding DLB disease modification. However, it is important to note that amyloid antibodies have only modest effects in leading to clinical improvement in Alzheimer’s disease and have known risks that would have to be balanced against the probability of acquiring clinically meaningful improvements.

Attenuating mitochondrial dysfunction has been a major theme of PD research, and a number of clinical trials are currently on-going targeting energy homeostasis. Nicotinamide riboside, a precursor of the essential mitochondrial oxidative phosphorylation cofactor, nicotinamide adenine dinucleotide (NAD+), is currently in Phase II clinical trial in PD [110]. Although no efficacy data is currently available, future indications of improvement in PD could suggest this therapy may also be effective in DLB given mitochondrial dysfunction is thought to be a feature of DLB. A recent study has indicated that terazosin, a drug that promotes ATP production through glycolysis, is associated with lower risk of developing DLB [111]. One could speculate that enhancing ATP production through glycolysis could compensate for diminished ATP production due to mitochondrial dysfunction, and may suggest this could be an effective treatment for DLB. However, it is important to emphasise that it is currently unclear whether mitochondrial dysfunction is a cause or consequence of DLB; thus, it is not clear whether improvement of mitochondrial function will be truly disease-modifying. Nevertheless, the close association between mitochondrial dysfunction and Lewy body disease more broadly would suggest this direction is worth further exploration.

Although proteopathy and mitochondrial dysfunction are common features of DLB, relatively few studies have sought to understand pathological changes that could underlie both. A plausible factor underlying both features is deficiencies in the autophagy-lysosomal pathway, as this would plausibly lead to concentration-dependent aggregation of α-synuclein and mitochondrial dysfunction through reduced mitophagy [112]. Although enhancing autophagy has been suggested as a therapeutic strategy in DLB, supported by positive results from pre-clinical studies using autophagy promoting agents such as rapamycin, it remains untested [113–115]. Given the evidence that autophagy is deficient in DLB and that its restoration can attenuate cell death and phenotype in vivo, future studies may wish to investigate whether rapamycin or other autophagy enhancers are beneficial in the treatment of DLB. In our recent study, we identified a number of commonly used drugs, such as celecoxib and memantine, which activate autophagy and could thus be repurposed to target autophagy in DLB [116].

A key aspiration must be to understand the mechanisms that underlie the full spectrum of pathological changes observed in DLB, with a view to targeting all pathological changes rather than one or two in isolation. As noted above, a number of Lewy body disease risk genes encode lysosomal enzymes responsible for the degradation of sphingolipids. Although most of these risk genes have been identified in PD, perhaps due to larger numbers of samples increasing study power, *GBA1* encoding glucocerebrosidase is amongst the strongest risk factors for DLB [117]. A Phase II clinical trial targeting glucocerebrosidase with the mucolytic ambroxol has recently commenced [118]. Given that a number of other sphingolipid catabolic enzymes have been implicated in Lewy body disease more broadly, including *SMPD1*, *GALC* and *ARSA*, future studies may wish to determine whether targeting the function of their protein products, or the prosaposins that activate many of these enzymes, is a useful therapeutic strategy in DLB.

## Conclusions

The clinical management of DLB is currently framed around targeting specific symptom domains to improve patient and caregiver quality of life. Such interventions are modestly effective but there is a pressing need for disease-modifying drugs to treat the underlying cause of the disease and alleviate symptoms more holistically. A key barrier to the development of disease-modifying drugs is lack of understanding about the cause of DLB and, by extension, appropriate methods through which it can be attenuated. A number of therapies are currently being explored to treat either proteopathy, mitochondrial dysfunction or deficient autophagy in isolation. However, a more complete understanding of the pathological processes that underlie the full spectrum of changes observed in the DLB brain is likely to herald the development of more effective therapies by treating the disease holistically, rather than particular manifestations.

## Data Availability

No datasets were generated or analysed during the current study.

## References

[1] Outeiro TF, Koss DJ, Erskine D, Walker L, Kurzawa-Akanbi M, Burn D, et al. Dementia with lewy bodies: an update and outlook. Mol Neurodegener. 2019;14(1):5. 30665447 10.1186/s13024-019-0306-8 PMC6341685

[2] McKeith IG, Boeve BF, Dickson DW, Halliday G, Taylor JP, Weintraub D, et al. Diagnosis and management of dementia with lewy bodies: fourth consensus report of the DLB consortium. Neurology. 2017;89(1):88–100. 28592453 10.1212/WNL.0000000000004058 PMC5496518

[3] Vann Jones SA, O’Brien JT. The prevalence and incidence of dementia with lewy bodies: a systematic review of population and clinical studies. Psychol Med. 2014;44(4):673–83. 23521899 10.1017/S0033291713000494

[4] Porter A, Christian L, Tipton P, Graff-Radford N, Day G, editors. Diagnostic performance and clinical applications of a commercial Alpha-synuclein seed amplification assay in a tertiary care memory clinic (S1. 010). Neurology: AAN Enterprises. 2024.

[5] Tiraboschi P, Salmon DP, Hansen LA, Hofstetter RC, Thal LJ, Corey-Bloom J. What best differentiates lewy body from alzheimer’s disease in early-stage dementia? Brain. 2006;129(Pt 3):729–35. 16401618 10.1093/brain/awh725

[6] Fedorova TD, Knudsen K, Horsager J, Hansen AK, Okkels N, Gottrup H, et al. Dopaminergic dysfunction is more symmetric in dementia with lewy bodies compared to parkinson’s disease. J Parkinsons Dis. 2023;13(4):515–23. 37212074 10.3233/JPD-230001 PMC10357144

[7] Goldman JG, Goetz CG, Brandabur M, Sanfilippo M, Stebbins GT. Effects of dopaminergic medications on psychosis and motor function in dementia with lewy bodies. Mov Disord. 2008;23(15):2248–50. 18823039 10.1002/mds.22322

[8] McKeith IG, Ferman TJ, Thomas AJ, Blanc F, Boeve BF, Fujishiro H, et al. Research criteria for the diagnosis of prodromal dementia with lewy bodies. Neurology. 2020;94(17):743–55. 32241955 10.1212/WNL.0000000000009323 PMC7274845

[9] Kane JP, Surendranathan A, Bentley A, Barker SA, Taylor J-P, Thomas AJ, et al. Clinical prevalence of lewy body dementia. Alzheimers Res Ther. 2018;10:1–8. 29448953 10.1186/s13195-018-0350-6 PMC5815202

[10] O’Brien JT, Taylor J-P, Thomas A, Bamford C, Vale L, Hill S, et al. Improving the diagnosis and management of lewy body dementia: the DIAMOND-Lewy research programme including pilot cluster RCT. Programme Grants Appl Res. 2021;9(7):1–120. 34339153

[11] Gomes BF, Farris CM, Ma Y, Concha-Marambio L, Lebovitz R, Nellgård B, et al. α-Synuclein seed amplification assay as a diagnostic tool for parkinsonian disorders. Parkinsonism Relat Disord. 2023;117:105807. 37591709 10.1016/j.parkreldis.2023.105807

[12] Hoglinger GU, Adler CH, Berg D, Klein C, Outeiro TF, Poewe W, et al. A biological classification of parkinson’s disease: the synneurge research diagnostic criteria. Lancet Neurol. 2024;23(2):191–204. 38267191 10.1016/S1474-4422(23)00404-0

[13] Simuni T, Chahine LM, Poston K, Brumm M, Buracchio T, Campbell M, et al. A biological definition of neuronal alpha-synuclein disease: towards an integrated staging system for research. Lancet Neurol. 2024;23(2):178–90. 38267190 10.1016/S1474-4422(23)00405-2

[14] Kotzbauer PT, Trojanowski JQ, Lee VM-Y. Lewy body pathology in alzheimer’s disease. J Mol Neurosci. 2001;17:225–32. 11816795 10.1385/jmn:17:2:225

[15] Kalia LV, Berg D, Kordower JH, Shannon KM, Taylor JP, Cardoso F, et al. International Parkinson and movement disorder society viewpoint on biological frameworks of Parkinson’s disease: current status and future directions. Mov Disord. 2024;39(10):1710–5. 39250594 10.1002/mds.30007

[16] Ghanem S, Hawksworth J, Thom S, Hartanto AE, O’Neill J, Ponraj J et al. Seed-competent alpha-synuclein pathology in metachromatic leukodystrophy: the expanding spectrum of alpha-synucleinopathy in sphingolipidoses. BioRxiv. 2024;2024.08.09.607301.

[17] Hatton C, Ghanem SS, Koss DJ, Abdi IY, Gibbons E, Guerreiro R, et al. Prion-like alpha-synuclein pathology in the brain of infants with Krabbe disease. Brain. 2022;145(4):1257–63. 34999780 10.1093/brain/awac002 PMC9128812

[18] Coughlin DG, MacLeod KR, Middleton JS, Bozoki AC, Galvin JE, Irwin DJ, et al. Association of CSF α-Synuclein seeding amplification assay results with clinical features of possible and probable dementia with lewy bodies. Neurology. 2024;103(3):e209656. 39013126 10.1212/WNL.0000000000209656 PMC11238940

[19] Hall S, Orrù CD, Serrano GE, Galasko D, Hughson AG, Groveman BR, et al. Performance of αSynuclein RT-QuIC in relation to neuropathological staging of lewy body disease. Acta Neuropathol Commun. 2022;10(1):90. 35733234 10.1186/s40478-022-01388-7 PMC9219141

[20] Taylor J-P, McKeith IG, Burn DJ, Boeve BF, Weintraub D, Bamford C, et al. New evidence on the management of lewy body dementia. Lancet Neurol. 2020;19(2):157–69. 31519472 10.1016/S1474-4422(19)30153-X PMC7017451

[21] Schumacher J, Taylor JP, Hamilton CA, Firbank M, Cromarty RA, Donaghy PC, et al. In vivo nucleus basalis of meynert degeneration in mild cognitive impairment with lewy bodies. Neuroimage Clin. 2021;30:102604. 33711623 10.1016/j.nicl.2021.102604 PMC7972982

[22] Hatton C, Reeve A, Lax NZ, Blain A, Ng YS, El-Agnaf O, et al. Complex I reductions in the nucleus basalis of meynert in lewy body dementia: the role of lewy bodies. Acta Neuropathol Commun. 2020;8(1):103. 32646480 10.1186/s40478-020-00985-8 PMC7346628

[23] Hepp DH, Ruiter AM, Galis Y, Voorn P, Rozemuller AJ, Berendse HW, et al. Pedunculopontine cholinergic cell loss in hallucinating Parkinson disease patients but not in dementia with lewy bodies patients. J Neuropathol Exp Neurol. 2013;72(12):1162–70. 24226265 10.1097/NEN.0000000000000014

[24] Mori E, Ikeda M, Nagai R, Matsuo K, Nakagawa M, Kosaka K. Long-term donepezil use for dementia with lewy bodies: results from an open-label extension of phase III trial. Alzheimers Res Ther. 2015;7(1):5. 25713600 10.1186/s13195-014-0081-2 PMC4338564

[25] Wild R, Pettit T, Burns A. Cholinesterase inhibitors for dementia with lewy bodies. Cochrane Database Syst Rev. 2003;2003(3):CD003672. 12917981 10.1002/14651858.CD003672 PMC7389676

[26] Xu H, Habich A, Ferreira D, Elisabet L, Westman E, Eriksdotter M. Long-term effects of cholinesterase inhibitors and memantine on cognitive decline, cardiovascular events, and mortality in dementia with lewy bodies: an up to 10‐year follow‐up study. Alzheimer’s & Dementia. 2024. 10.1002/alz.14118 PMC11485406 39177108

[27] Tari PK, Parsons CG, Collingridge GL, Rammes G. Memantine: updating a rare success story in pro-cognitive therapeutics. Neuropharmacology. 2024;244:109737. 37832633 10.1016/j.neuropharm.2023.109737

[28] Kutzing MK, Luo V, Firestein BL. Protection from glutamate-induced excitotoxicity by memantine. Ann Biomed Eng. 2012;40(5):1170–81. 22203191 10.1007/s10439-011-0494-z PMC4217523

[29] Matsunaga S, Kishi T, Iwata N. Memantine for lewy body disorders: systematic review and meta-analysis. Am J Geriatr Psychiatry. 2015;23(4):373–83. 24406251 10.1016/j.jagp.2013.11.007

[30] Emre M, Tsolaki M, Bonuccelli U, Destée A, Tolosa E, Kutzelnigg A, et al. Memantine for patients with parkinson’s disease dementia or dementia with lewy bodies: a randomised, double-blind, placebo-controlled trial. Lancet Neurol. 2010;9(10):969–77. 20729148 10.1016/S1474-4422(10)70194-0

[31] Zweig YR, Galvin JE. Lewy body dementia: the impact on patients and caregivers. Alzheimers Res Ther. 2014;6:1–7. 25031635 10.1186/alzrt251 PMC4054937

[32] Armstrong MJ, Weintraub D. The case for antipsychotics in dementia with lewy bodies. Mov Disorders Clin Pract. 2017;4(1):32. 10.1002/mdc3.12383 PMC6353494 30713945

[33] Armstrong MJ, Weintraub D. The case for antipsychotics in dementia with lewy bodies. Mov Disorders Clin Pract. 2016;4(1):32. 10.1002/mdc3.12383 PMC6353494 30713945

[34] Sugawara Kikuchi Y, Shimizu T. Aripiprazole for the treatment of psychotic symptoms in patients with dementia with lewy bodies: a case series. Neuropsychiatr Dis Treat. 2019: 15:543–7. 10.2147/NDT.S189050 10.2147/NDT.S189050 PMC6390858 30863076

[35] Boylan L, Hirsch S. Motor worsening and tardive dyskinesia with aripiprazole in Lewy body dementia. BMJ Case Rep. 2009;2009. 10.1136/bcr.06.2008.0205 PMC3027689 21686897

[36] Horn S, Richardson H, Xie SX, Weintraub D, Dahodwala N. Pimavanserin versus quetiapine for the treatment of psychosis in parkinson’s disease and dementia with lewy bodies. Parkinsonism Relat Disord. 2019;69:119–24. 31751863 10.1016/j.parkreldis.2019.11.009 PMC7061324

[37] Rothenberg KG, McRae SG, Dominguez-Colman LM, Shutes-David A, Tsuang DW. Pimavanserin treatment for psychosis in patients with dementia with lewy bodies: A case series. Am J Case Rep. 2023;24:e939806–1. 37775968 10.12659/AJCR.939806 PMC10549935

[38] Borda MG, Jaramillo-Jimenez A, Oesterhus R, Santacruz JM, Tovar‐Rios DA, Soennesyn H, et al. Benzodiazepines and antidepressants: effects on cognitive and functional decline in alzheimer’s disease and lewy body dementia. Int J Geriatr Psychiatry. 2021;36(6):917–25. 33382911 10.1002/gps.5494

[39] O’Brien JT, McKeith IG, Thomas AJ, Bamford C, Vale L, Hill S, et al. Introduction of a management toolkit for lewy body dementia: A pilot Cluster-Randomized trial. Mov Disord. 2021;36(1):143–51. 32960456 10.1002/mds.28282

[40] Watts KE, Storr NJ, Barr PG, Rajkumar AP. Systematic review of Pharmacological interventions for people with lewy body dementia. Aging Ment Health. 2023;27(2):203–16. 35109724 10.1080/13607863.2022.2032601

[41] Nevels RM, Gontkovsky ST, Williams BE. Paroxetine—the antidepressant from hell? Probably not, but caution required. Psychopharmacol Bull. 2016;46(1):77. 27738376 10.64719/pb.4345 PMC5044489

[42] Molloy S, McKeith I, O’brien J, Burn D. The role of Levodopa in the management of dementia with lewy bodies. J Neurol Neurosurg Psychiatry. 2005;76(9):1200–3. 16107351 10.1136/jnnp.2004.052332 PMC1739807

[43] Lucetti C, Logi C, Del Dotto P, Berti C, Ceravolo R, Baldacci F, et al. Levodopa response in dementia with lewy bodies: a 1-year follow-up study. Parkinsonism Relat Disord. 2010;16(8):522–6. 20615745 10.1016/j.parkreldis.2010.06.004

[44] Hershey LA, Coleman-Jackson R. Pharmacological management of dementia with lewy bodies. Drugs Aging. 2019;36:309–19. 30680679 10.1007/s40266-018-00636-7 PMC6435621

[45] Murata M, Odawara T, Hasegawa K, Iiyama S, Nakamura M, Tagawa M, et al. Adjunct Zonisamide to Levodopa for DLB parkinsonism: a randomized double-blind phase 2 study. Neurology. 2018;90(8):e664–72. 29367449 10.1212/WNL.0000000000005010 PMC5818167

[46] Murata M, Odawara T, Hasegawa K, Kajiwara R, Takeuchi H, Tagawa M, et al. Effect of Zonisamide on parkinsonism in patients with dementia with lewy bodies: a phase 3 randomized clinical trial. Parkinsonism Relat Disord. 2020;76:91–7. 31982288 10.1016/j.parkreldis.2019.12.005

[47] Mizukami K. Autonomic dysfunction in dementia with lewy bodies: focusing on cardiovascular and respiratory dysfunction. Psychiatry Clin Neurosciences Rep. 2023;2(3):e129. 10.1002/pcn5.129 PMC11114397 38867816

[48] Guidi L, Evangelisti S, Siniscalco A, Lodi R, Tonon C, Mitolo M. Non-pharmacological treatments in lewy body disease: a systematic review. Dement Geriatr Cogn Disord. 2023;52(1):16–31. 36977397 10.1159/000529256

[49] Connors MH, Quinto L, McKeith I, Brodaty H, Allan L, Bamford C, et al. Non-pharmacological interventions for lewy body dementia: a systematic review. Psychol Med. 2018;48(11):1749–58. 29143692 10.1017/S0033291717003257 PMC6088773

[50] Elder GJ, Taylor J-P. Transcranial magnetic stimulation and transcranial direct current stimulation: treatments for cognitive and neuropsychiatric symptoms in the neurodegenerative dementias? Alzheimers Res Ther. 2014;6:1–11. 25478032 10.1186/s13195-014-0074-1 PMC4255638

[51] Takahashi S, Mizukami K, Yasuno F, Asada T. Depression associated with dementia with lewy bodies (DLB) and the effect of somatotherapy. Psychogeriatrics. 2009;9(2):56–61. 19604326 10.1111/j.1479-8301.2009.00292.x

[52] Elder GJ, Firbank MJ, Kumar H, Chatterjee P, Chakraborty T, Dutt A, et al. Effects of transcranial direct current stimulation upon attention and visuoperceptual function in lewy body dementia: a preliminary study. Int Psychogeriatr. 2016;28(2):341–7. 26250473 10.1017/S1041610215001180 PMC4720143

[53] Elder GJ, Colloby SJ, Firbank MJ, McKeith IG, Taylor J-P. Consecutive sessions of transcranial direct current stimulation do not remediate visual hallucinations in lewy body dementia: a randomised controlled trial. Alzheimers Res Ther. 2019;11:1–13. 30658705 10.1186/s13195-018-0465-9 PMC6339360

[54] Yamaguchi Y, Matsuoka K, Ueda J, Takada R, Takahashi M, Kiuchi K, et al. The effect of electroconvulsive therapy on psychiatric symptoms of dementia with lewy bodies. J Neuropsychiatry Clin Neurosci. 2016;28:e66.

[55] Morikawa F, Kobayashi R, Murayama T, Fukuya S, Tabata K, Fujishiro H, et al. Evaluating electroconvulsive therapy for dementia with lewy bodies, including the prodromal stage: A retrospective study on safety and efficacy. Int J Geriatr Psychiatry. 2024;39(12):e70020. 39608804 10.1002/gps.70020

[56] Gratwicke J, Zrinzo L, Kahan J, Peters A, Brechany U, McNichol A, et al. Bilateral nucleus basalis of meynert deep brain stimulation for dementia with lewy bodies: a randomised clinical trial. Brain Stimul. 2020;13(4):1031–9. 32334074 10.1016/j.brs.2020.04.010

[57] Konno T, Ross OA, Puschmann A, Dickson DW, Wszolek ZK. Autosomal dominant parkinson’s disease caused by SNCA duplications. Parkinsonism Relat Disord. 2016;22(Suppl 1Suppl 1):S1–6. 26350119 10.1016/j.parkreldis.2015.09.007 PMC4820832

[58] Campelo C, Silva RH. Genetic variants in SNCA and the risk of sporadic parkinson’s disease and clinical outcomes: A review. Parkinsons Dis. 2017;2017:4318416. 28781905 10.1155/2017/4318416 PMC5525082

[59] Mittal S, Bjornevik K, Im DS, Flierl A, Dong X, Locascio JJ, et al. beta2-Adrenoreceptor is a regulator of the alpha-synuclein gene driving risk of parkinson’s disease. Science. 2017;357(6354):891–8. 28860381 10.1126/science.aaf3934 PMC5761666

[60] Spillantini MG, Schmidt ML, Lee VM, Trojanowski JQ, Jakes R, Goedert M. Alpha-synuclein in lewy bodies. Nature. 1997;388(6645):839–40. 9278044 10.1038/42166

[61] Parkkinen L, O’Sullivan SS, Collins C, Petrie A, Holton JL, Revesz T, et al. Disentangling the relationship between lewy bodies and nigral neuronal loss in parkinson’s disease. J Parkinsons Dis. 2011;1(3):277–86. 23939308 10.3233/JPD-2011-11046 PMC4196643

[62] Gomez-Tortosa E, Newell K, Irizarry M, Albert M, Growdon J, Hyman BT. Clinical and quantitative pathologic correlates of dementia with lewy bodies. Neurology. 1999;53(6):1284. 10522886 10.1212/wnl.53.6.1284

[63] Erskine D, Thomas AJ, Attems J, Taylor JP, McKeith IG, Morris CM, et al. Specific patterns of neuronal loss in the pulvinar nucleus in dementia with lewy bodies. Mov Disord. 2017;32(3):414–22. 28059471 10.1002/mds.26887

[64] Bernstein HG, Johnson M, Perry RH, LeBeau FE, Dobrowolny H, Bogerts B, et al. Partial loss of parvalbumin-containing hippocampal interneurons in dementia with lewy bodies. Neuropathology. 2011;31(1):1–10. 20487308 10.1111/j.1440-1789.2010.01117.x

[65] Khundakar AA, Hanson PS, Erskine D, Lax NZ, Roscamp J, Karyka E, et al. Analysis of primary visual cortex in dementia with lewy bodies indicates GABAergic involvement associated with recurrent complex visual hallucinations. Acta Neuropathol Commun. 2016;4:1–18. 27357212 10.1186/s40478-016-0334-3 PMC4928325

[66] Martin WRW, Younce JR, Campbell MC, Racette BA, Norris SA, Ushe M, et al. Neocortical lewy body pathology parallels parkinson’s dementia, but not always. Ann Neurol. 2023;93(1):184–95. 36331161 10.1002/ana.26542 PMC10321306

[67] Roberts RF, Wade-Martins R, Alegre-Abarrategui J. Direct visualization of alpha-synuclein oligomers reveals previously undetected pathology in parkinson’s disease brain. Brain. 2015;138(6):1642–57. 25732184 10.1093/brain/awv040 PMC4614141

[68] Sekiya H, Tsuji A, Hashimoto Y, Takata M, Koga S, Nishida K, et al. Discrepancy between distribution of alpha-synuclein oligomers and Lewy-related pathology in parkinson’s disease. Acta Neuropathol Commun. 2022;10(1):133. 36068646 10.1186/s40478-022-01440-6 PMC9450240

[69] Muntané G, Ferrer I, Martinez-Vicente M. α-synuclein phosphorylation and Truncation are normal events in the adult human brain. Neuroscience. 2012;200:106–19. 22079575 10.1016/j.neuroscience.2011.10.042

[70] Ramalingam N, Jin S-X, Moors TE, Fonseca-Ornelas L, Shimanaka K, Lei S, et al. Dynamic physiological α-synuclein S129 phosphorylation is driven by neuronal activity. Npj Parkinson’s Disease. 2023;9(1):4. 36646701 10.1038/s41531-023-00444-w PMC9842642

[71] Ghanem SS, Majbour NK, Vaikath NN, Ardah MT, Erskine D, Jensen NM, et al. α-Synuclein phosphorylation at Serine 129 occurs after initial protein deposition and inhibits seeded fibril formation and toxicity. Proc Natl Acad Sci. 2022;119(15):e2109617119. 35353605 10.1073/pnas.2109617119 PMC9169642

[72] Zhang S, Zhu R, Pan B, Xu H, Olufemi MF, Gathagan RJ, et al. Post-translational modifications of soluble α-synuclein regulate the amplification of pathological α-synuclein. Nat Neurosci. 2023;26(2):213–25. 36690898 10.1038/s41593-022-01239-7 PMC10103650

[73] Chia R, Sabir MS, Bandres-Ciga S, Saez-Atienzar S, Reynolds RH, Gustavsson E, et al. Genome sequencing analysis identifies new loci associated with lewy body dementia and provides insights into its genetic architecture. Nat Genet. 2021;53(3):294–303. 33589841 10.1038/s41588-021-00785-3 PMC7946812

[74] Irwin DJ, Grossman M, Weintraub D, Hurtig HI, Duda JE, Xie SX, et al. Neuropathological and genetic correlates of survival and dementia onset in synucleinopathies: a retrospective analysis. Lancet Neurol. 2017;16(1):55–65. 27979356 10.1016/S1474-4422(16)30291-5 PMC5181646

[75] Walker L, Stefanis L, Attems J. Clinical and neuropathological differences between parkinson’s disease, parkinson’s disease dementia and dementia with lewy bodies - current issues and future directions. J Neurochem. 2019;150(5):467–74. 30892688 10.1111/jnc.14698

[76] Schumacher J, Gunter JL, Przybelski SA, Jones DT, Graff-Radford J, Savica R, et al. Dementia with lewy bodies: association of alzheimer pathology with functional connectivity networks. Brain. 2021;144(10):3212–25. 34114602 10.1093/brain/awab218 PMC8634124

[77] Montine TJ, Phelps CH, Beach TG, Bigio EH, Cairns NJ, Dickson DW, et al. National Institute on Aging–Alzheimer’s association guidelines for the neuropathologic assessment of alzheimer’s disease: a practical approach. Acta Neuropathol. 2012;123:1–11. 22101365 10.1007/s00401-011-0910-3 PMC3268003

[78] Toledo JB, Abdelnour C, Weil RS, Ferreira D, Rodriguez-Porcel F, Pilotto A, et al. Dementia with lewy bodies: impact of co‐pathologies and implications for clinical trial design. Alzheimer’s Dement. 2023;19(1):318–32. 36239924 10.1002/alz.12814 PMC9881193

[79] Soto C, Estrada LD. Protein misfolding and neurodegeneration. Arch Neurol. 2008;65(2):184–9. 18268186 10.1001/archneurol.2007.56

[80] Johnston JA, Ward CL, Kopito RR. Aggresomes: a cellular response to misfolded proteins. J Cell Biol. 1998;143(7):1883–98. 9864362 10.1083/jcb.143.7.1883 PMC2175217

[81] Pickrell AM, Youle RJ. The roles of PINK1, parkin, and mitochondrial fidelity in parkinson’s disease. Neuron. 2015;85(2):257–73. 25611507 10.1016/j.neuron.2014.12.007 PMC4764997

[82] Rajkumar AP, Bidkhori G, Shoaie S, Clarke E, Morrin H, Hye A, et al. Postmortem cortical transcriptomics of lewy body dementia reveal mitochondrial dysfunction and lack of neuroinflammation. Am J Geriatr Psychiatry. 2020;28(1):75–86. 31327631 10.1016/j.jagp.2019.06.007

[83] Reeve AK, Park TK, Jaros E, Campbell GR, Lax NZ, Hepplewhite PD, et al. Relationship between mitochondria and alpha-synuclein: a study of single substantia Nigra neurons. Arch Neurol. 2012;69(3):385–93. 22410447 10.1001/archneurol.2011.2675

[84] Erskine D, Reeve AK, Polvikoski T, Schaefer AM, Taylor RW, Lax NZ, et al. Lewy body pathology is more prevalent in older individuals with mitochondrial disease than controls. Acta Neuropathol. 2020;139(1):219–21. 31781911 10.1007/s00401-019-02105-w PMC6942000

[85] Ng YS, Lax NZ, Blain AP, Erskine D, Baker MR, Polvikoski T, et al. Forecasting stroke-like episodes and outcomes in mitochondrial disease. Brain. 2022;145(2):542–54. 34927673 10.1093/brain/awab353 PMC9014738

[86] Erskine D, Patterson L, Alexandris A, Hanson PS, McKeith IG, Attems J, et al. Regional levels of physiological alpha-synuclein are directly associated with lewy body pathology. Acta Neuropathol. 2018;135(1):153–4. 29134319 10.1007/s00401-017-1787-6

[87] Ludtmann MHR, Angelova PR, Horrocks MH, Choi ML, Rodrigues M, Baev AY, et al. alpha-synuclein oligomers interact with ATP synthase and open the permeability transition pore in parkinson’s disease. Nat Commun. 2018;9(1):2293. 29895861 10.1038/s41467-018-04422-2 PMC5997668

[88] Choi ML, Chappard A, Singh BP, Maclachlan C, Rodrigues M, Fedotova EI, et al. Pathological structural conversion of alpha-synuclein at the mitochondria induces neuronal toxicity. Nat Neurosci. 2022;25(9):1134–48. 36042314 10.1038/s41593-022-01140-3 PMC9448679

[89] Amin J, Erskine D, Donaghy PC, Surendranathan A, Swann P, Kunicki AP, et al. Inflammation in dementia with lewy bodies. Neurobiol Dis. 2022;168:105698. 35314318 10.1016/j.nbd.2022.105698

[90] King E, O’Brien JT, Donaghy P, Morris C, Barnett N, Olsen K, et al. Peripheral inflammation in prodromal alzheimer’s and lewy body dementias. J Neurol Neurosurg Psychiatry. 2018;89(4):339–45. 29248892 10.1136/jnnp-2017-317134 PMC5869446

[91] Amin J, Holmes C, Dorey RB, Tommasino E, Casal YR, Williams DM, et al. Neuroinflammation in dementia with lewy bodies: a human post-mortem study. Transl Psychiatry. 2020;10(1):267. 32747635 10.1038/s41398-020-00954-8 PMC7400566

[92] Tomlinson JJ, Shutinoski B, Dong L, Meng F, Elleithy D, Lengacher NA, et al. Holocranohistochemistry enables the visualization of α-synuclein expression in the murine olfactory system and discovery of its systemic anti-microbial effects. J Neural Transm. 2017;124:721–38. 28477284 10.1007/s00702-017-1726-7 PMC5446848

[93] Labrie V, Brundin P. Alpha-synuclein to the rescue: immune cell recruitment by alpha-synuclein during Gastrointestinal infection. J Innate Immun. 2017;9(5):437–40. 28866688 10.1159/000479653 PMC5607025

[94] Tulisiak CT, Mercado G, Peelaerts W, Brundin L, Brundin P. Can infections trigger alpha-synucleinopathies? Prog Mol Biol Transl Sci. 2019;168:299–322. 31699323 10.1016/bs.pmbts.2019.06.002 PMC6857718

[95] Gan-Or Z, Dion PA, Rouleau GA. Genetic perspective on the role of the autophagy-lysosome pathway in Parkinson disease. Autophagy. 2015;11(9):1443–57. 26207393 10.1080/15548627.2015.1067364 PMC4590678

[96] Longobardi A, Catania M, Geviti A, Salvi E, Vecchi ER, Bellini S, et al. Autophagy markers are altered in alzheimer’s disease, dementia with lewy bodies and frontotemporal dementia. Int J Mol Sci. 2024;25(2):1125. 38256197 10.3390/ijms25021125 PMC10816165

[97] Higashi S, Moore DJ, Minegishi M, Kasanuki K, Fujishiro H, Kabuta T, et al. Localization of MAP1-LC3 in vulnerable neurons and lewy bodies in brains of patients with dementia with lewy bodies. J Neuropathology Experimental Neurol. 2011;70(4):264–80. 10.1097/NEN.0b013e318211c86a 21412173

[98] Shahmoradian SH, Lewis AJ, Genoud C, Hench J, Moors TE, Navarro PP, et al. Lewy pathology in parkinson’s disease consists of crowded organelles and lipid membranes. Nat Neurosci. 2019;22(7):1099–109. 31235907 10.1038/s41593-019-0423-2

[99] Mazzetti S, De Leonardis M, Gagliardi G, Calogero AM, Basellini MJ, Madaschi L, et al. Phospho-HDAC6 gathers into protein aggregates in parkinson’s disease and atypical parkinsonisms. Front Neurosci. 2020;14:624. 32655357 10.3389/fnins.2020.00624 PMC7324673

[100] Lansbury P. The sphingolipids clearly play a role in parkinson’s disease, but nature has made it complicated. Mov Disord. 2022;37(10):1985–9. 36087026 10.1002/mds.29204

[101] Erskine D, Koss D, Korolchuk VI, Outeiro TF, Attems J, McKeith I. Lipids, lysosomes and mitochondria: insights into lewy body formation from rare Monogenic disorders. Acta Neuropathol. 2021;141(4):511–26. 33515275 10.1007/s00401-021-02266-7 PMC7952289

[102] Knupp J, Martinez-Montanes F, Van Den Bergh F, Cottier S, Schneiter R, Beard D, et al. Sphingolipid accumulation causes mitochondrial dysregulation and cell death. Cell Death Differ. 2017;24(12):2044–53. 28800132 10.1038/cdd.2017.128 PMC5686345

[103] Settembre C, Fraldi A, Jahreiss L, Spampanato C, Venturi C, Medina D, et al. A block of autophagy in lysosomal storage disorders. Hum Mol Genet. 2008;17(1):119–29. 17913701 10.1093/hmg/ddm289

[104] Pagano G, Taylor KI, Anzures-Cabrera J, Marchesi M, Simuni T, Marek K, et al. Trial of prasinezumab in early-stage parkinson’s disease. N Engl J Med. 2022;387(5):421–32. 35921451 10.1056/NEJMoa2202867

[105] Pagano G, Taylor KI, Anzures Cabrera J, Simuni T, Marek K, Postuma RB et al. Prasinezumab slows motor progression in rapidly progressing early-stage parkinson’s disease. Nat Med. 2024;30(4):1096–1103. 10.1038/s41591-024-02886-y 10.1038/s41591-024-02886-y PMC11031390 38622249

[106] McFarthing K, Buff S, Rafaloff G, Pitzer K, Fiske B, Navangul A et al. Parkinson’s disease drug therapies in the clinical trial pipeline: 2024 update. J Parkinson’s Disease. 2024. 10.3233/JPD-240272 10.3233/JPD-240272 PMC11307066 39031388

[107] Cummings J. Anti-amyloid monoclonal antibodies are transformative treatments that redefine alzheimer’s disease therapeutics. Drugs. 2023;83(7):569–76. 37060386 10.1007/s40265-023-01858-9 PMC10195708

[108] Van Dyck CH, Swanson CJ, Aisen P, Bateman RJ, Chen C, Gee M, et al. Lecanemab in early alzheimer’s disease. N Engl J Med. 2023;388(1):9–21. 36449413 10.1056/NEJMoa2212948

[109] Sims JR, Zimmer JA, Evans CD, Lu M, Ardayfio P, Sparks J, et al. Donanemab in early symptomatic alzheimer disease: the Trailblazer-ALZ 2 randomized clinical trial. JAMA. 2023;330(6):512–27. 37459141 10.1001/jama.2023.13239 PMC10352931

[110] Berven H, Kverneng S, Sheard E, Søgnen M, Af Geijerstam SA, Haugarvoll K, et al. NR-SAFE: a randomized, double-blind safety trial of high dose nicotinamide riboside in parkinson’s disease. Nat Commun. 2023;14(1):7793. 38016950 10.1038/s41467-023-43514-6 PMC10684646

[111] Hart A, Aldridge G, Zhang Q, Narayanan NS, Simmering JE. Association of terazosin, doxazosin, or Alfuzosin use and risk of dementia with lewy bodies in men. Neurology. 2024;103(2):e209570. 38896813 10.1212/WNL.0000000000209570 PMC11226317

[112] Themistokleous C, Bagnoli E, Parulekar R, Muqit MM. Role of autophagy pathway in parkinson’s disease and related genetic neurological disorders. J Mol Biol. 2023;435(12):168144. 37182812 10.1016/j.jmb.2023.168144

[113] Moors TE, Hoozemans JJ, Ingrassia A, Beccari T, Parnetti L, Chartier-Harlin M-C, et al. Therapeutic potential of autophagy-enhancing agents in parkinson’s disease. Mol Neurodegeneration. 2017;12:1–18. 10.1186/s13024-017-0154-3 PMC5267440 28122627

[114] Khan MR, Yin X, Kang S-U, Mitra J, Wang H, Ryu T, et al. Enhanced mTORC1 signaling and protein synthesis in pathologic α-synuclein cellular and animal models of parkinson’s disease. Sci Transl Med. 2023;15(724):eadd0499. 38019930 10.1126/scitranslmed.add0499

[115] Malagelada C, Jin ZH, Jackson-Lewis V, Przedborski S, Greene LA. Rapamycin protects against neuron death in in vitro AndIn vivo models of parkinson’s disease. J Neurosci. 2010;30(3):1166–75. 20089925 10.1523/JNEUROSCI.3944-09.2010 PMC2880868

[116] Kataura T, Sedlackova L, Sun C, Kocak G, Wilson N, Banks P, et al. Targeting the autophagy-NAD axis protects against cell death in Niemann-Pick type C1 disease models. Cell Death Dis. 2024;15(5):382. 38821960 10.1038/s41419-024-06770-y PMC11143325

[117] Vergouw LJ, van Steenoven I, van de Berg WD, Teunissen CE, van Swieten JC, Bonifati V, et al. An update on the genetics of dementia with lewy bodies. Parkinsonism Relat Disord. 2017;43:1–8. 28734699 10.1016/j.parkreldis.2017.07.009

[118] Chwiszczuk LJ, Breitve MH, Kirsebom B-EB, Selnes P, Fløvig JC, Knapskog A-B, et al. The ANeED study–ambroxol in new and early dementia with lewy bodies (DLB): protocol for a phase IIa multicentre, randomised, double-blinded and placebo-controlled trial. Front Aging Neurosci. 2023;15:1163184. 37304077 10.3389/fnagi.2023.1163184 PMC10250712

